# Predictors of mTICI 2c/3 over 2b in patients successfully recanalized with mechanical thrombectomy

**DOI:** 10.1002/acn3.51935

**Published:** 2023-11-06

**Authors:** Richard Wang, Jing Huang, Alireza Mohseni, Meisam Hoseinyazdi, Apoorva Kotha, Omar Hamam, Julie Gudenkauf, Hye Young Heo, Mehreen Nabi, Judy Huang, Fernando Gonzalez, Golnoosh Ansari, Mahla Radmard, Licia Luna, Justin Caplan, Risheng Xu, Vivek Yedavalli

**Affiliations:** ^1^ Department of Radiology and Radiological Sciences, Division of Neuroradiology Johns Hopkins School of Medicine Baltimore Maryland USA; ^2^ School of Nursing Johns Hopkins University Baltimore Maryland USA; ^3^ Department of Neurosurgery Johns Hopkins School of Medicine Baltimore Maryland USA

## Abstract

**Objective:**

For patients presenting with acute ischemic stroke (AIS) caused by large vessel occlusions (LVO), mechanical thrombectomy (MT) is the treatment standard of care in eligible patients. Modified Thrombolysis in Cerebral Infarction (mTICI) grades of 2b, 2c, and 3 are all considered successful reperfusion; however, recent studies have shown achieving mTICI 2c/3 leads to better outcomes than mTICI 2b. This study aims to investigate whether any baseline preprocedural or periprocedural parameters are predictive of achieving mTICI 2c/3 in successfully recanalized LVO patients.

**Methods:**

We conducted a retrospective multicenter cohort study of consecutive patients presenting with AIS caused by a LVO from 1 January 2017 to 1 January 2023. Baseline and procedural data were collected through chart review. Univariate and multivariate analysis were applied to determine significant predictors of mTICI 2c/3.

**Results:**

A total of 216 patients were included in the study, with 159 (73.6%) achieving mTICI 2c/3 recanalization and 57 (26.4%) achieving mTICI 2b recanalization. We found that a higher groin puncture to first pass time (OR = 0.976, 95%CI: 0.960–0.992, *p* = 0.004), a higher first pass to recanalization time (OR = 0.985, 95%CI: 0.972–0.998, *p* = 0.029), a higher admission NIHSS (OR = 0.949, 95%CI: 0.904–0.995, *p* = 0.031), and a lower age (OR = 1.032, 95%CI: 1.01–1.055, *p* = 0.005) were associated with a decreased probability of achieving mTICI 2c/3.

**Interpretation:**

A lower groin puncture to first pass time, a lower first pass to recanalization time, a lower admission NIHSS, and a higher age were independent predictors of mTICI 2c/3 recanalization.

## Introduction

Acute ischemic stroke (AIS) is a key cause of mortality and morbidity worldwide.^1^ Up to 30% of AIS cases are caused by anterior circulation large vessel occlusions (LVOs), which have been shown to cause disproportionately worse outcomes than other sites of occlusion.^2^, ^3^ For these patients, mechanical thrombectomy (MT) has become the standard of care for up to 24 hours in patients deemed eligible.^4^, ^5^

The extent of reperfusion achieved after MT is typically assessed with the modified Thrombolysis in Cerebral Infarction (mTICI) scale. Historically, mTICI grades of 2b, 2c, and 3 have all been considered successful recanalization^5^; however, recent literature suggests that patients who achieve a mTICI grade of 2c or 3—considered excellent recanalization—have superior outcomes than those with 2b recanalization.^6^, ^7^, ^8^, ^9^ The superior outcomes associated with mTICI 2c/3 underscores the importance of investigating prognostic biomarkers predictive for excellent recanalization.

The objective of the current study is to therefore investigate which baseline preprocedural or periprocedural parameters are predictive of excellent recanalization (defined as mTICI 2c/3) in successfully recanalized LVO patients (defined as mTICI 2b/2c/3).

## Materials and Methods

The corresponding author had full access to all the data in the study and takes responsibility for its integrity and the data analysis.

### Study population

We performed a retrospective multicenter cohort study of all patients presenting with AIS caused by a large vessel occlusion from 1 January 2017 to 1 January 2023. This study was approved by our institutional review board (protocol code JHU‐IRB00269637) and complies with the Health Insurance Portability and Accountability Act. Inclusion criteria were patients with AIS secondary to anterior circulation LVO (defined as distal intracranial ICA, M1, and proximal M2 segments of the middle cerebral artery (MCA)) who were evaluated within 24 h of symptom onset and successfully treated with MT to achieve mTICI 2b, 2c, or 3 recanalization. The mTICI was determined by the performing neuro‐interventionalist. Patients with intracranial hemorrhage on baseline imaging were excluded from the study. Study participants were then categorized into either a mTICI 2b or a combined mTICI 2c/3 cohort for comparative analysis.

### Data collection

Baseline, clinical, and time parameter data were abstracted from review of electronic medical records. Baseline labs and vitals were based on first available values on initial admission. For further details, refer to our preliminary paper which used the same methodology.^10^ ASPECTS score was assessed by an experienced neuroradiologist (Dr. Vivek Yedavalli, 6 years of experience).

### Study outcomes

The primary outcome measure was achieving mTICI 2c/3 recanalization.

### Statistics

The data were managed and analyzed using IBM SPSS statistics (Statistical Package for Social Sciences) software version 26, IBM Corp., Chicago, IL, USA, 2013 and Microsoft Excel. All qualitative data were described as *n*, %, and compared using chi‐squared and Fisher's exact tests. All quantitative data except for time parameter data were reported as mean ± SD (standard deviation) and compared using independent *t*‐tests. Time parameter data were reported as median (IQR) with *p*‐value determined by a nonparametric test. Each baseline variable was independently assessed through univariate analysis. Those variables with a *p*‐value < 0.1, as well as age and sex, were then entered in a logistic regression model (backward stepwise method) to determine the independent factors associated with mTICI 2c/3. *p*‐values of < 0.05 were considered significant.

## Results

Two hundred sixteen total patients were included in this study, of which 159 (73.6%) achieved mTICI 2c/3 recanalization and 57 (26.4%) achieved mTICI 2b recanalization.

The baseline characteristics of patients who achieved mTICI 2c/3 were compared with those who achieved mTICI 2b (Table 1). The mean ± SD age of the studied cases was 68.5 ± 15.1 years. Notably, patients who achieved mTICI 2c/3 were more likely to have a lower admission NIHSS score than those who achieved mTICI 2b, which trended toward significance (14.7 ± 6.7 vs. 16.8 ± 7.6, *p* = 0.053).

**Table 1:** Descriptive statistics among studied cases and comparison according to mTICI grade.

Variables	All cases (*N* = 216)	mTICI grade		*p*‐value*
		2c/3 (*N* = 159)	2b (*N* = 57)	
Age (years)	68.5 ± 15.1	69.6 ± 14.7	65.4 ± 15.7	0.070
Sex (*n*, %)				
Male	90 (41.7)	67 (42.1)	23 (40.4)	0.814
Female	126 (58.3)	92 (57.9)	34 (59.6)	
Race (*n*, %)				
White/Caucasian	117 (54.2)	88 (55.3)	29 (50.9)	0.835
African	84 (38.9)	60 (37.7)	24 (42.1)	
Others	15 (6.9)	11 (6.9)	4 (7.0)	
BMI (kg/m^2^)	28.8 ± 8.2	28.1 ± 7.0	30.7 ± 10.8	0.099
BMI grade (*n*, %)				
<30.0	136 (63.3)	106 (66.7)	30 (53.6)	0.080
≥30.0	79 (36.7)	53 (33.3)	26 (46.4)	
SBP (mmHg)	151.7 ± 29.6	152.4 ± 29.5	149.7 ± 30.1	0.557
DBP (mmHg)	88.8 ± 21.6	89.4 ± 21.6	87.2 ± 21.9	0.499
HR (beats/minute)	85.9 ± 20.9	86.0 ± 20.8	85.5 ± 21.2	0.880
RR (cycles/minute)	18.4 ± 4.3	18.2 ± 3.6	19.1 ± 5.8	0.182
SPO_2_ (%)	97.8 ± 2.3	97.7 ± 2.4	98.2 ± 1.9	0.103
Glucose (mg/dL)	137.0 ± 64.3	135.0 ± 56.3	142.5 ± 82.8	0.450
Sodium	138.6 ± 3.6	138.7 ± 3.5	138.4 ± 3.9	0.698
Potassium	4.2 ± 0.6	4.2 ± 0.7	4.0 ± 0.5	0.114
BUN	19.0 ± 9.6	19.0 ± 9.5	18.9 ± 9.9	0.995
Creatinine	1.1 ± 0.6	1.1 ± 0.6	1.1 ± 0.4	0.879
Hematocrit	39.4 ± 5.1	39.3 ± 5.0	39.8 ± 5.5	0.468
Hemoglobin (gm/dL)	12.8 ± 1.8	12.8 ± 1.8	13.0 ± 1.8	0.528
WBC	8.8 ± 3.7	8.6 ± 3.8	9.1 ± 3.6	0.447
Platelets	231.4 ± 83.6	229.6 ± 88.6	236.2 ± 68.1	0.611
Admission NIHSS	15.2 ± 7.0	14.7 ± 6.7	16.8 ± 7.6	0.053
ASPECTS	8.7 ± 1.7	8.8 ± 1.7	8.6 ± 1.6	0.446
Subtype of ischemic stroke per TOAST criteria (*n*, %)				
Large artery atherosclerosis	32 (14.9)	19 (12.0)	13 (22.8)	0.220
Cardioembolic	120 (55.8)	92 (58.2)	28 (49.1)	
Small‐vessel occlusion	0 (0)	0 (0)	0 (0)	
Stroke of other determined etiology	12 (5.6)	8 (5.1)	4 (7.0)	
Stroke of undetermined etiology	51 (23.7)	39 (24.7)	12 (21.1)	
Occlusion laterality (*n*, %)				
Right	102 (47.2)	79 (77.5)	23 (22.5)	0.226
Left	114 (52.8)	80 (50.3)	34 (59.6)	

Data presented as Mean ± SD unless mentioned otherwise.

BMI, body mass index; BUN, blood urea nitrogen; DBP, diastolic blood pressure; HR, heart rate; RR, respiratory rate; NIHSS, National Institutes of Health Stroke Scale; SBP, systolic blood pressure.

* Significant (<0.05).

Time parameter and related intervention data between the mTICI 2c/3 and 2b cohorts were also compared (Table 2). A lower groin puncture to first pass time (22.0 [16.0–29.0] min vs. 27.0 [18.0–38.5] min, *p* = 0.019) and a lower first pass to recanalization time (5.0 [2.0–18.0] min vs. 14.0 [3.0–38.5] min, *p* = 0.029) were significantly associated with mTICI 2c/3.

**Table 2:** Comparison of intervention and time parameter data between the mTICI 2b and mTICI 2c/3 cohorts.

Variables	All cases (*N* = 216)	mTICI grade		*p*‐value
		2c/3 (*N* = 159)	2b (*N* = 57)	
Symptom onset to door time (min) (median, IQR)	169.5 (61.5, 633.3)	185.0 (60,0, 622.0)	138.0 (62.0, 726.0)	0.686
Door to CT time (min)	25.0 (14.0, 38.3)	25.0 (14.0, 38.3)	25.0 (15.0, 38.8)	0.751
Door to needle time (min)	57.5 (41.8, 78.0)	57.0 (42.0, 79.0)	59.0 (39.0, 77.0)	0.967
Door to groin puncture time (min)	152.5 (122.0, 211.8)	154.0 (122.0, 206.0)	152.0 (123.5, 247.5)	0.544
Groin puncture to first pass time (min)	23.0 (16.0, 31.0)	22.0 (16.0, 29.0)	27.0 (18.0, 38.5)	0.019*
First pass to recanalization time (mins)	6.0 (3.0, 22.0)	5.0 (2.0, 18.0)	14 (3.0, 38.5)	0.029*
Number of passes (Mean ± SD)	1.6 ± 1.0	1.5 ± 1.1	1.7 ± 0.9	0.274
Type of anesthesia (*n*, %)				
General	195 (90.7)	145 (91.8)	50 (87.7)	0.366
MAC	20 (9.3)	13 (8.2)	7 (12.3)	
Type of thrombectomy device (*n*, %)				
Direct aspiration	121 (56.8)	97 (61.8)	24 (42.9)	0.042*
Stent retriever	23 (10.8)	14 (8.9)	9 (16.1)	
Combined	69 (32.4)	46 (29.3)	23 (41.1)	
IV tPA administered (*n*, %)	75 (34.7)	52 (32.7)	23 (40.4)	0.298

Data presented as median (IQR) unless mentioned otherwise.

tPA, tissue plasminogen activator.

* Significant (<0.05).

After adjusting for potential confounders, age, admission NIHSS, groin puncture to first pass time, and first pass to recanalization time were significantly associated with mTICI 2c/3 (Table 3). Specifically, a higher groin puncture to first pass time (OR = 0.976, 95%CI: 0.960–0.992, *p* = 0.004), a higher first pass to recanalization time (OR = 0.985, 95%CI: 0.972–0.998, *p* = 0.029), a higher admission NIHSS (OR = 0.949, 95%CI: 0.904–0.995, *p* = 0.031), and a lower age (OR = 1.032, 95%CI: 1.01–1.055, *p* = 0.005) were associated with a decreased probability of achieving mTICI 2c/3.

**Table 3:** Multivariate logistic regression of studied variables in predicting mTICI 2c/3 in all patients.

Variable	OR	95% C.I.		*p*‐value
		Lower	Upper	
Age	1.032	1.010	1.055	0.005*
Admission NIHSS	0.949	0.904	0.995	0.031*
Groin puncture to first pass time	0.976	0.960	0.992	0.004*
First pass to recanalization time	0.985	0.972	0.998	0.029*

C.I., confidence interval; NIHSS, National Institutes of Health Stroke Scale; OR, odds ratio.

* Significant (<0.05).

## Discussion

In this multicenter, retrospective study, we found that a lower groin puncture to first pass time, a lower first pass to recanalization time, a lower admission NIHSS, and a higher age were independent predictors of excellent recanalization.

MT is the treatment standard of care in many patients presenting with AIS secondary to LVOs. While mTICI 2b, 2c, and 3 recanalization are all considered successful reperfusion, recent literature shows that patients who achieve mTICI 2c/3 recanalization have better outcomes than those who only achieve mTICI 2b recanalization.^6^, ^7^, ^8^, ^9^ Which factors determine whether successfully recanalized patients achieve excellent recanalization is still underexplored. Our study aimed to fill this knowledge gap by examining potential prognostic biomarkers predictive of excellent recanalization following endovascular treatment.

In our study, a lower groin puncture to first pass time was an independent predictor of excellent recanalization. The mTICI 2c/3 group had a median groin puncture to first pass time of 22.0 [16.0–29.0] min as compared to the mTICI 2b group average of 27.0 [18.0–38.5] min, representing a roughly 5‐min difference between the two cohorts. Thus, minimizing the time needed to deploy the clot retrieval device after groin puncture is of key importance, regardless of the type of device used. While a previous study showed that shorter MT procedure times are associated with mTICI 3 over 2b,^11^ this is the first study to our knowledge that specifically identified groin puncture to first pass time as being associated with excellent recanalization. Initiating MT through a groin puncture is an invasive procedure that may destabilize the patient, and prolonging the time needed until device deployment can make this deployment less effective. For example, prolonged surgery has been shown to damage the blood–brain barrier and allow entry of inflammatory cytokines as well as cause widespread microglia activation, both of which increase the risk of neuroinflammation.^12^, ^13^, ^14^ Delays after groin puncture gives more time for this neuroinflammation to occur, which may in turn hinder both the guiding of the catheter and the deployment of the clot retrieval device, reducing the extent of reperfusion achieved. Minimizing the time needed to perform the procedure is also overall advantageous, as faster recanalization may improve the quality of reperfusion, such as by reducing ischemic damage or decreasing the likelihood of distal clot embolization.^10^, ^11^, ^15^, ^16^

Alongside a shorter groin puncture to first pass time, our study found that a lower first pass to recanalization time was also an independent predictor of excellent recanalization. The mTICI 2c/3 group had a median first pass to recanalization time of 5.0 [2.0–18.0] min as compared to the mTICI 2b group average of 14.0 [3.0–38.5] min, representing a nearly 10‐min difference between the two cohorts. Importantly, the number of passes needed to achieve recanalization was not significantly different between the two study cohorts (*p* = 0.274). This suggests that to achieve excellent recanalization, it is important to minimize delays in retrieving the clot after the clot retrieval device is deployed. One possible reason may be that the device causes stress to the vasculature nearby the occlusion, inducing local inflammatory changes that makes achieving and maintaining recanalization more difficult.^17^, ^18^, ^19^ Of note, several prior studies have advocated for waiting several minutes following stent retriever deployment to allow for better integration between the device and the clot, thereby achieving superior clot removal.^20^, ^21^, ^22^ Indeed, many proceduralists wait for up to several minutes following stent deployment before beginning the clot removal process.^23^, ^24^ While there may be a benefit to allocate some amount of waiting time when using stent retrievers to improve clot retrieval, our study shows that excessive waiting will ultimately lead to worse recanalization regardless of the thrombectomy device used. Future studies should clarify the optimal balance between maximizing device‐clot integration and minimizing unnecessary delays of clot removal to achieve maximum recanalization.

Lower admission NIHSS was also an independent predictor of mTICI 2c/3 recanalization. It has been widely shown that stroke severity as determined by admission NIHSS is a crucial predictor of poststroke clinical outcomes, with higher NIHSS levels associated with greater functional impairment and mortality.^25^, ^26^, ^27^, ^28^ There is also some evidence that lower admission NIHSS is associated with greater mTICI score following MT.^6^, ^29^ However, this is the first study to our knowledge that shows that a lower admission NIHSS can act as an independent predictor of mTICI 2c/3 over 2b within the subset of successfully recanalized patients. There are several possible reasons for this. First, patients who present with a more severe stroke may have a larger and/or more invasive blood clot that is more challenging to remove, resulting in less complete recanalization of the occluded vessel(s).^30^, ^31^ Second, higher admission NIHSS is associated with worse collateral status,^32^, ^33^, ^34^ which in turn is associated with less successful reperfusion following endovascular treatment.^35^, ^36^ Third, patients with more severe stroke symptoms are more likely to already have extensive and/or irreversible ischemic brain damage than those with milder symptoms, whether due to the severity of the clot or a delay in their evaluation, limiting the amount of reperfusion that can be achieved through MT.

Surprisingly, our study also found that older age was an independent predictor of excellent recanalization. Older age is typically associated with worse poststroke outcomes, with older patients having worse functional recovery, greater morbidity, and higher mortality after a stroke event.^37^, ^38^, ^39^, ^40^ While there is still some debate on the efficacy of mechanical thrombectomy in the elderly population, available studies on the topic suggest that these patients also have worse post‐ MT functional outcomes than those who are younger.^41^, ^42^, ^43^ The relationship between age and post‐MT radiographic outcomes like mTICI, however, has been much less studied. One possible explanation for why older age may increase mTICI 2c/3 recanalization over mTCI 2b is treatment selection bias. Due to concerns of performing an invasive procedure like MT in elderly patients, the treatment team may only select older patients with favorable clinical and radiographic characteristics, like low clot burden and high collateral status^44^; these patients will be much more likely to achieve excellent recanalization. In contrast, nearly all younger patients will be considered a candidate for mechanical thrombectomy, even those with unfavorable characteristics that reduce the likelihood of achieving excellent recanalization. With an increasing older adult population, further research on the impact of age on stroke treatment outcomes is needed.

There are several study design limitations that should be considered when interpreting these results. First, our study has the inherent limitations of a retrospective approach. We aimed to minimize this by only including consecutive patients from two comprehensive stroke centers within our larger hospital enterprise. Second, the mTICI scores were self‐adjudicated by the operating physician, and thus may not be completely standardized. However, all mTICI scores used in the study were adjudicated by experienced neurointerventionists with years of training, all within the same institution. Third, our sample size was imbalanced between the 2c/3 and 2b patient cohorts (73.6% of the total study population vs. 26.4%, respectively), which is a potential source of bias. Prior studies have shown that more patients generally achieve mTICI 2c or 3 than 2b following MT.^6^, ^8^ As an example, in a 2017 study that included 1933 successfully recanalized patients, 63.8% of the total study population was in the 2c/3 cohort.^6^ Another 2021 study that included 149 successfully recanalized patients had 81.2% of their study population in the 2c/3 cohort.^8^ Our sample size differences therefore lie within the ranges of prior investigations. Fourth, identifying periprocedural factors that are independent predictors of excellent recanalization (i.e., groin puncture to first pass time) may be less useful for selecting patients for MT compared to identifying predictive baseline characteristics (i.e., age or NIHSS score), as the former is only determined after the procedure has started. However, they are still useful for maximizing outcomes after the patient has been selected for MT and as quality improvement markers for the procedure itself.

In conclusion, we report that lower groin puncture to first pass time, lower first pass to recanalization time, a lower admission NIHSS, and older age were independent predictors of mTICI 2c/3 recanalization. Understanding these findings may help providers better maximize the likelihood of achieving excellent recanalization in patients presenting with LVOs. Additional prospective studies must be conducted to further elucidate the impact of these parameters on achieving excellent recanalization.

## Author Contributions

*Idea generation, primary writing, editing*: Richard Wang. *Stats, secondary writing*: Jing Huang. *Stats, editing*: Alireza Mohseni. *Editing*: Meisam Hoseinyazdi, Apoorva Kotha, Omar Hamam, Julie Gudenkauf, Hye Young Heo, Mehreen Nabi, Judy Huang, Golnoosh Ansari, Mahla Radmard, Licia Luna, Justin Caplan, Risheng Xu. *Idea generation, primary writing, stats, editing*: Vivek Yedavalli.

## Conflict of Interest Statement

The authors do not have any conflict of interest to disclose except for Dr. Vivek Yedavalli, who serves as a consultant for MRIOnline (Cincinnati, OH, USA), RAPID (IschemaView, Menlo Park, CA, USA), and editorial board of Frontiers in Radiology.
